# Homocysteine, Vitamin B12 and Folate Level: Possible Risk Factors in the Progression of Chronic Heart and Kidney Disorders

**DOI:** 10.2174/1573403X19666230209111854

**Published:** 2023-07-05

**Authors:** Aditi Mohan, Ravi Kumar, Vivek Kumar, Mahendra Yadav

**Affiliations:** 1 Amity Institute of Biotechnology, Amity University, Noida Sector 125, Uttar Pradesh, India;; 2 Department of Life Sciences, Bharathiyar University, Coimbatore, Tamil Nadu 641046, India;; 3 Delhi Pharmaceutical Sciences and Research University, New Delhi 110017, India;; 4 Narayan Institute of Pharmacy, Gopal Narayan Singh University, Sasaram, Rohtas, Bihar 821305, India

**Keywords:** Homocysteine, Vitamin B12, chronic kidney disease, cardiovascular system, cobalamin, ESRD

## Abstract

Cobalamin is an essential molecule for humans; it is exceptionally important for various body functions, including deoxyribonucleic acid synthesis and cellular energy production. Vegans are more vulnerable to vitamin B12 deficiency than natives with moderate consumption of animal dietary supplements or people with inadequate nutritional patterns. However, the long-term effects of sub-medical deficiency have not been thoroughly studied, but they may have a negative impact on the cardiovascular system, pregnancy outcomes, and vascular, renal, cognitive, bone, and eye health. Alongside the statin remedy, that is a powerful approach for CVD prevention. Another approach is related to the B nutrition substitution remedy with folic acid, and vitamins B6 and B12 are extensively practised nowadays. There is a tremendous interest in plasma homocysteine (tHcy) as a cardiovascular hazard factor. However, current research in the field of its prevention is more inclined toward confirming the benefit of tHcy-reducing remedy with vitamin B12. Thus, while folic acid fortification is primarily aimed at reducing neural-tube defects, it may also play a significant role in the primary prevention of CVD by lowering tHcy. Folate and B-vitamins play important roles in CVD prevention and nutrition policy implementation. Patients affected with Chronic Kidney Disease (CKD) or end-stage Stage Renal Disease (ESRD) experience a tremendous cardiovascular threat that may also further lead to death. As a result, routine monitoring of vitamin B12 levels is likely to be beneficial for the early detection and treatment of metabolic vitamin B12 deficiency, as well as the prevention of heart-related diseases.

## 1 INTRODUCTION

The prevalence of chronic kidney disease (CKD) is an issue for global public health. Vitamin B12 is an essential nutrient that functions as a coenzyme in metabolic processes, remethylation of homocysteine to methionine, and converts methyl malonyl-CoA to succinyl-CoA. It aids in the metabolism of homocysteine, which occurs *via* remethylation to methionine or transsulfuration to cysteine [1]. Homocysteine mediated extended lipid peroxidation and production of free radicals species results in acute endothelial disorder and inflammation, similarly accelerating atherosclerotic methods persuading cardiovascular problems. Heart-associated problems are majorly connected with the extended degree of homocysteine, which plays a liberal function in endothelial damage [2]. In general use, low concentrations of vitamin B12 and hyperhomocysteinemia are the fundamental challenges for cardiovascular disorders. Unusual vascular shape and features had been additionally discovered in sufferers with vitamin B12 deficiency. However, in affiliation with metabolic vitamin B12 deficiency, their carotid intima-media thickness and branchial flow-mediated dilation had been crucially worsened compared to those with normal vitamin B12. Considering many of these facts, it is probably beneficial to increase plasma vitamin B12 and homocysteine levels owing to their close affiliation with carotid intima-media thickness [3]. Homocysteine metabolism depends on vitamins B12 and folate. Hyperhomocysteinemia has been linked to endothelial dysfunction and cardiovascular disease [1].

In contrast to hemorrhagic strokes, ischemic strokes are currently more common, and their incidence is higher in older people. However, diet poses a manageable risk factor for strokes. The metabolic network that integrates dietary cues with biosynthesis, epigenetics, and redox homeostasis comprises B vitamins [4]. Vegetarians and the aged population are at greater risk of vitamin B12 deficiency. Among the aged, metabolic vitamin B12 deficiency happens in approximately 20% of the natives, out of which >60% are because of food-cobalamin malabsorption syndrome caused by gastrointestinal problems [3]. Also noteworthy is that those with stage 3 CKD are more likely to die from CVD than patients with ESRD. Additionally, people with renal and cardiovascular disease have a substantially greater mortality risk than those with just one condition [4].

Deficiency associated with the absence of intrinsic elements ends in severe neurological impairments and unsafe anemia. Consequently, such people need clinical attention comprising vitamin B12 injections [5]. A deficiency of vitamin B12 leads to the build-up of methylmalonic acid (MMA) because of the inactivation of methylmalonyl-CoA mutase; consequently, the increased concentration of Hcy and MMA is dangerous. Therefore the cells deliver these metabolites into the circulatory system. Thus serum Hcy and MMA acquired from intracellular enzymatic reactions are cell markers of the B12 profile [6]. Some CVD risk factors simultaneously play a role in the advancement of CKD and vice versa, providing more evidence of a limited cross-talk that may speed up both processes. Since there are no particular medications to prevent CVD in this group, the delay of ESRD remains a fundamental objective of CKD therapy [4]. This is because a link between CKD and CVD exists from the early stages of the disease. Due to their hemodynamic/antihypertensive, anti-inflammatory/antifibrotic, and angiotensin II receptor blockade actions, ACE inhibitors and angiotensin II receptor blockers are the backbone of pharmaceutical therapy for CKD, attempting to halt the development of ESRD [4]. Due to the increased rise in cardiovascular mortality, those with chronic kidney disease (CKD) or end-stage renal disease (ESRD) have a significantly shorter life expectancy than people with adequate renal function [7]. Patients on chronic hemodialysis have a 10 to 15 fold higher risk of death than the native population, mostly due to cardiovascular disease development (CVD). However, this increased risk of cardiac disease has been noted since the earliest stages of CKD [8]. The “homocysteine hypothesis” was developed in response to the finding that patients with significantly elevated homocysteine levels due to congenital homocysteine metabolism degradation are more likely to progress to a severe type of atherosclerosis. Although the people with ESRD and CKD had higher homocysteine levels, the role of hyperhomocysteinemia as a risk factor for cardiovascular disease and mortality remains unclear and warrants additional research. Although the exact mechanism of action is still unknown, studies have shown that the B vitamins (folate, B6, and B12) may play a role in reducing the risk of cardiovascular disease. This is because lowering Hcy levels has significantly reduced the risk of coronary artery disease and stroke. However, there is ongoing debate regarding how these nutritional supplements affect the secondary prevention of CVD or strokes [9]. No research has probably examined the relationship between the consumption of these vitamins and the risk of heart failure. However, Hcy concentrations above the median (11.8 mmol/L for males and 11.1 mmol/L for women) are associated with a two-fold increased risk [10].

Stringent blood pressure management, strict glycemic control, and quitting smoking are among the preventative interventions that can decrease the onset of CKD [11]. However, information explaining the connection between homocysteine and CKD is still lacking [12]. Prior cross-sectional investigations found a link between high homocysteine levels and poor renal function, but these studies had their limitations, such as being restricted to a small cohort or a specific population from the Far East.

A study of medical data from 17,010 participants, 67% of whom were men, between the years 2000 and 2014 revealed significant differences between the four quartiles of homocysteine concentrations and estimated glomerular filtration rate (eGFR)—the greater the homocysteine concentration, the lower the eGFR (p 0.0001) [13]. In addition, a special uremic toxin called lanthionine was discovered in recent research on dialysis patients. This toxin may help to cause hyperhomocysteinemia, which is seen in uremic individuals [14]. Hhcy only substantially mediates the effect of renal impairment on atherosclerosis [15]. Impaired renal metabolism and decreased renal excretion induce hyperhomocysteinemia (Hhcy) in roughly 85% of CKD patients [14]. The transformation of homocysteine (Hcy) into methionine depends on folic acid (FA), a synthetic form of vitamin B9. Insufficient FA intake results in inadequate conversion, which raises Hcy levels [16]. In patients with end-stage renal illness, hhcy is recognized as a reliable predictor of cardiovascular morbidity and death. Hhcy has a pathogenic effect on the key mechanisms driving the development of vascular injury. According to research, Hhcy raises the risk of endothelial damage, inflammation, cardiovascular disease (CVD), stroke, and CKD [8]. Another explanation for FA's impact on endothelial function is that it has been demonstrated to enhance endothelial function without decreasing Hcy. In certain randomized studies, the function of FA and Hhcy in the development of CKD and CVD was recently re-evaluated [16]. Current discussion surrounds whether any positive effects of FA treatment in the general population and in CKD patients should be attributed to their direct effect or to a decrease in Hhcy. It is possible to consider FA with or without methylcobalamin supplementation as a suitable supplementary treatment in individuals with CKD while awaiting the findings of confirmatory trials.

## 2 MATERIALS AND METHODS

The literature review was conducted for the evaluation of treatment options for risk factors in metabolic vitamin B12 insufficiency and likely for the prevention of chronic heart and kidney disorder.

We searched several electronic databases using systematic review methods. The literature that was found in searches was critically reviewed, both in terms of quality reporting and usefulness to decision-makers. The literature was searched dated from 2011/01/01 to 2022/9/31 using Google Scholar, PubMed, and another database. This review incorporates the search results of 1628 publications after preliminary screening on PubMed, Google Scholar, and online resources. 1012 publications and documents were then shortlisted after an abstract and methodology screening. Fig. (**1**) depicts the flowchart of the selection of the final article. A total of 462 articles and documents were excluded after the full-text screening, of which 57 were written in languages other than English, and 206 failed to provide significant findings for Homocysteine, vitamin B12, and folate levels; as potential risk factors in the progression of CVD and CKD.

### 2.1 Vitamin B_12_, Folate, and the Methionine Remethylation Cycle-biochemistry, Pathways, and Regulation

There are several different forms of vitamin B12, usually known as cobalamin (Cbl), including cyano-, methyl-deoxyadenosyl-, and hydroxy-cobalamin. To prevent the progression of end-stage renal disease (ESRD) and lower cardiovascular mortality, reasoned action is required. Considering the use of dialysis, annual mortality rates continue to be above 20%, with cardiovascular disease accounting for more than half of all fatalities [17]. Peripheral artery disease (PAD), which is more prevalent in individuals with CKD and is linked to lower limb amputations and higher mortality, is another issue that is rising [18]. There are several different forms of vitamin B12, usually known as cobalamin (Cbl), including cyano-, methyl-deoxyadenosyl-, and hydroxy-cobalamin. The cyno- form, utilized as supplement, is generally present in diets in minute amounts. Methionine synthase plays a major role in the synthesis of purines and pyrimidines. When folate and methylcobalamin are present as cofactors in the process, the methyl group of methlytetrahydrofolate is converted to homocysteine, releasing methionine and tetrahydrofolate. Megaloblastic anemia is caused by any disruption of this mechanism or a deficiency in vitamin B12 [19]. Methionine synthase reductase catalyzes the transformation of methionine into S-adenosylmethionine (SAM) and S-adenosylhomocysteine (SAH). SAM is one of the most significant methyl group donor and is essential for the breakdown of a variety of amino acids and fatty acids [19, 20].

**Fig. (2):**
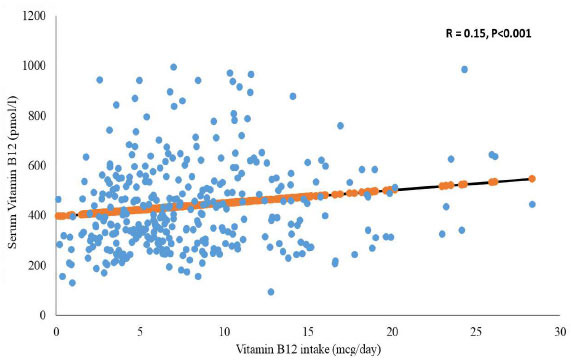
Correlation between vitamin B12 intake and vitamin B12 levels.

Transcobalamin (TC), the protein that plasma vitamin B12 attaches to, transports around 80% of it to inactive TCI (refer to haptocorrin). While the active transport protein for vitamin B12 transcobalamin ll (TCll) is expected to carry around 205 of the vitamins in the circulation. [21]. Another type is holo-transcobalamin (holo-TC), which is still linked to cobalamin and serves as a carrier of vitamin B12 in every cell. The inadequacy of TCl, however, may be the cause of a lower blood concentration of cobalamin when TCl and vitamin B12 levels are both appropriate [22]. However, further research is needed to determine the intermediate level of its concentration [23]. Homocysteine (Hcy) and methylmalonic acid (MMA) are frequently used as indicators of vitamin B12 insufficiency [15]. The occurrence of the sub-clinical deficit has been highlighted by their measurement, but its effects have not yet been fully understood [23]. Increased tHcy levels indicated a deficiency in folate, vitamin B6, and vitamin B12 as well, but methylmalonic acid is suggested to be a marker of vitamin B12 metabolism [7, 24]. There was a significant difference in vitamin B12 consumption between the vitamin-B12 sufficient and vitamin-B12 insufficient groups (7.0 mcg/day compared to 3.9 mcg/day; *p* = 0.01). Moreover, Spearman's correlation test revealed a significant positive relationship between vitamin B12 intake and vitamin B12 serum concentration (r = 0.15, *p* < 0.05; Fig. **2**).

Environmental or physiological factors may also confound these biological markers [25]. For instance, the use of certain drugs, polymorphism in methylenetetrahydrofolate reductase (MTHFR), or any disability in renal structure can be concluded as potential factors responsible for the elevation of the serum tHcy concentration [16]. In the older population, plasma MMA concentration rises due to renal inadequacy [16]. Numerous researchers have shown that assessing plasma vitamin B12 levels along with MMA quantifying is the most appropriate approach for assessing cobalamin status among natives [13]. Nonetheless, the threshold above which further testing of cobalamin should be done is still disputed. According to studies, people with increased MMA levels would be overlooked if a lower range of normal (200 ng/L or 147 pmol/L) was chosen [26]. Therefore, a higher level (500 ng/L or 370 pmol/L) accounts for patients with raised MMA levels. However, many people need further MMA testing, which may show results that are within the normal range. Studies that evaluated the use of holo-TC as a risk marker for vitamin B12 concentration found that it was comparable to plasma cobalamin levels in terms of specificity and sensitivity. However, the predicted value for identifying cobalamin deficiency is improved when vitamin B12 is combined with it [26].

Additionally, it's critical to remember that people with CKD may experience adverse effects from high B12 levels. This has to do with cyanide metabolism, which is aberrant in CKD patients because of the reduced glomerular filtrate. The most popular type of B12 supplement, cyanocobalamin, is converted into active methylcobalamin by releasing trace quantities of cyanide. Normally, cyanide and methylcobalamin bond with one another to form cyanocobalamin. However, decreased cyanide clearance in CKD patients inhibits cyanocobalamin from being converted into the active form, making incorporation into this form less efficient at lowering Hcy levels. Additionally, cyanocobalamin supplementation in excess can generate cyanide ions that are not eliminated and hasten the development of problems in CKD patients [22].

Me-Cbl and Ado-Cbl are the most prevalent forms of vitamin B12 that are frequently used as cell coenzymes [27]. Cobalt interacts with the 5,6-dimethylimidazole nucleotide base (DMB) molecule on the bottom side of the ring. Me-Cbl functions as a cofactor for methionine synthase and converts homocysteine to methionine with the help of vitamin B6 and folate [27]. This vital action occurs in the cytoplasm, and a vitamin cofactor deficit may cause an increase in serum Hcy levels [28]. This pathway is required for the replenishment of the methyl cofactor S-adenosylmethionine (SAM), and any disruption results in a shortage that harms the mechanism of DNA. Several neurological causes are mostly to blame for vitamin B12 deficiency [28]. Myeline sheath production in neurons relies heavily on the metabolism of fatty acids, and the depletion of Ado-Cbl in the neurons caused myeline sheath synthesis to decline, resulting in defective neuronal transmission [13]. The interactions between Cbl and neurotrophic molecules, including myelinolytic Tumor Necrosis Factor (TNF), interleukins, and epidermal growth factors, are primarily responsible for the crucial function of Cbl in neuropathological features [29]. The neurotoxic effects of Hcy on synaptic receptors are apparent. Some provitamins, including Cn-Cbl and H-Cbl, need to be activated beforehand by cofactors like Me-Cbl and Ado-Cbl for the cell to use them. Despite being first identified as a potential anti-pernicious anemia agent, Cn-Cbl is the cobalamin isoform [29].

Cobalamin also aids in the body's elimination of potentially harmful chemicals [30]. A minimal amount of cobalamin in blood flow is frequently detected as Cn-Cbl due to its interaction with cyanide residues. H-Cbl is another well-known type of physiological intermediate. Other corrinoids, which are assumed to be non-vitamin analogs, can bind with solid carriers, which makes them less accessible for interacting with vitamin forms and has a clear antinutrient effect [31, 32]. The isoforms of Cbl undergo dealkylation, declination, and reduction inside the cell to help in its metabolism inside the peroxisomes, after which it is released in one of the two cell compartments as the coenzymes Me-Cbl and Ado-Cbl depending on their mitochondrial propensity [33]. This process is crucial for triggering the provitamin forms. However, other corrinoids' inability to perform vitamin activities is mainly caused by the lower ligand's high affinity for cobalt, which prevents peroxisome activation [34].

### 2.2 Role of Methylenetetrahydrofolate Reductase (MTHFR) in the Metabolism of Vitamin B12

The B vitamins, including vitamin B12, are necessary cofactors for many enzymes for achieving body metabolic function [35]. Animal-based products are primary source of vitamin B12, such as meat, fish, lamb, eggs, and other dairy products. Although a gut-residing bacterium in humans, namely *Escherichia coli*, can produce cobalamin, the amount is insufficient to keep on par with the body's demands [36]. Various chaperons, molecular receptors, and transporter proteins safeguard vitamin B12 and further facilitate its absorption in the gastrointestinal tract and renal reabsorption for its proper distribution and retention. An intrinsic glycoprotein factor (IF) aids in its absorption [37].

Homocysteine is more rapidly methylated owing to the primary circulating form of folic acid, 5-methyltetrahydro folate reductase (MTHFR). Homocysteine levels above 10 ng and methylmalonic acid concentrations above 210 nM can be used to detect vitamin B12 deficiency [38]. As we grow and develop, our metabolism and absorption of different vitamins also alter. In India, more than 20% of the older population suffers from inadequate vitamin B12. This is either due to ill absorption or atrophic gastritis, resulting in alterations in gastric emptying and reduced secretion of intrinsic factors [36]. Individuals with high levels of homocysteine in the plasm are recommended to elevate the intake of products with a high level of folate or folic acid supplements to aid in reducing serum homocysteine concentration. However, treatments for such deficiencies involve intramuscular injection and oral or nasal supplementation of vitamin B12 [38].

Consuming food-grade vitamin B12, naturally found in foods like meat, eggs, and poultry bonded to protein, creates a complex [39]. This cobalamin-protein complex (Cbl-P) enters the stomach, where food enzymes like HCL and pepsinogen release the cobalamin from the animal protein [40]. The resulting Cbl liberation forms the cobalamin-R protein complex (Cbl-R), which travels to the colon. R-protein is a specialized protein released by the salivary gland and parietal cells [40]. When cobalamin reaches the intestinal system, a unique glycoprotein known as an intrinsic factor that is secreted by parietal cells in the stomach can bind to cobalamin. However, the intrinsic factor cannot survive in the stomach's acidic environment [9].

**Fig. (3):**
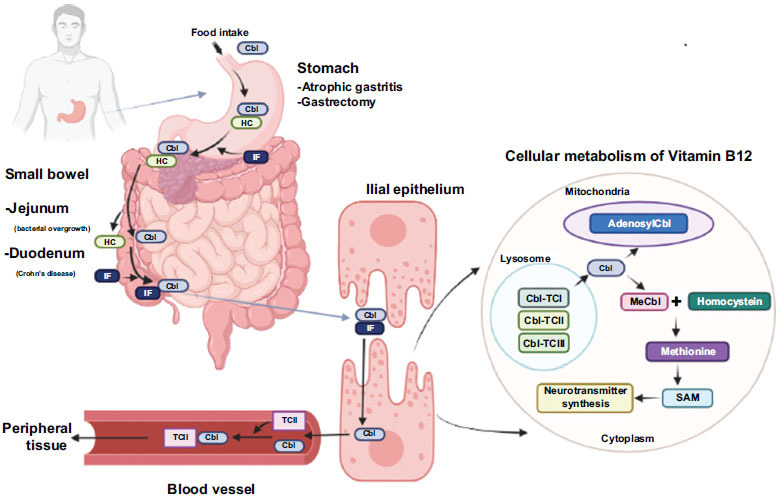
Cellular processing and trafficking mechanism of dietary Vitamin B12 in humans.

The pancreatic hydrolytic enzyme breaks down the Cbl-R complex in the intestinal tract, releasing cobalamin [36]. In order to create a cobalamin-intrinsic factor complex, the free cobalamin can now bond with the intrinsic factor (Cbl-IF) in Fig. (**3**) [9]. The mucosal layer of the cell's Cbl-IF, which is present, is subsequently bound by a receptor called cubilin, causing it to be endocytosed [18]. The complex is further broken when it is successfully endocytosed at the mucosal layer, releasing free cobalamin. Once in the bloodstream, the freed cobalamin joins forces with transcobalamin l, ll, or lll (TCl, TCll, TClll) to form a cobalamin-transcobalamin complex that travels to its intended destination [35]. Contrary to transcobalamin-l and transcobalamin-ll, cobalamin-transcobalamin-l demonstrated its exceptional capacity to circulate cobalamin throughout the entire body (Fig. **3**). While in circulation, cobalamin binds separately to each type of transcobalamin. Once within the target cell, the Cbl-TCll is endocytosed and quickly cleaves, releasing the cobalamin. In the target cell, the free cobalamin is subsequently converted into its two active forms in humans, methylcobalamin (Me-Cbl) and adenosylcobalamin (Adenosyl-Cbl). Methyl-Cbl, on the other hand, prefers the cytoplasm of the cell, whereas Adenosyl-Cbl gravitates toward the mitochondria. Methionine synthase and Me-Cbl work together to help methylate homocysteine into methionine. S-adenosyl methionine (SAM), which functions as a neurotransmitter in the brain, is further transformed from this methionine [35].

The 5,10-Methylenetetrahydrofolate reductase (MTH FR), a critical enzyme in the metabolism of folate and Hcy, produces the most important circulating form of folate, 5-MTHF [36]. The Hcy-methionine pathway has several gene polymorphisms that have been demonstrated to induce Hhcy, suggesting that these genetic variants may also play a role in a variety of multi-factorial problems with Hcy that are highly prevalent in the general population [36]. Despite the fact that there are other MTHFR gene variations, the single nucleotide polymorphisms at positions 677 (MTHFR 677C>T), 1298 (MTHFR 1298A>C), 1317 (MTH homocysteine plasma FR 1317T>C), and 1793 (MTHFR 1793G>A) are the best described and understood varieties [36]. The MTHFR gene has been found to have 15 rare variants that are linked to severe enzyme deficit and one common mutation, C677T, that is linked to a lesser enzyme deficit, according to research by Goyette *et al.* [20, 41]. The replacement of Cys with Arg, Arg with Glu, and Arg with Cys at 1015, 167, and 1081 bp, respectively, were the additional compound heterozygous mutations at exons 1, 5, and 6. The 59 bp deletion that caused the loss of 19 amino acids between 653 and 939 bp was recognized as the splice-site mutation [41]. Pregnant women with the MTHFR 677TT genotype had lower serum folate levels (p = 0.042) and higher Hcy levels (p = 0.003), according to a multivariate study by Aline Barnabe *et al.* [41].

Comparison of subjects with homocysteine plasma levels that are equal to or higher than 15 mol/L to those with homocysteine plasma levels that are lower than 15 mol/L and homocysteine plasma levels equal to or greater than 12 mol/L to men with homocysteine plasma levels less than 12 mol/L and women with homocysteine plasma levels equal to or greater than 10 mol/L to women with homocysteine plasma levels less than 10 mol/L is shown in Tables **1** and **2**.

**Fig. (4):**
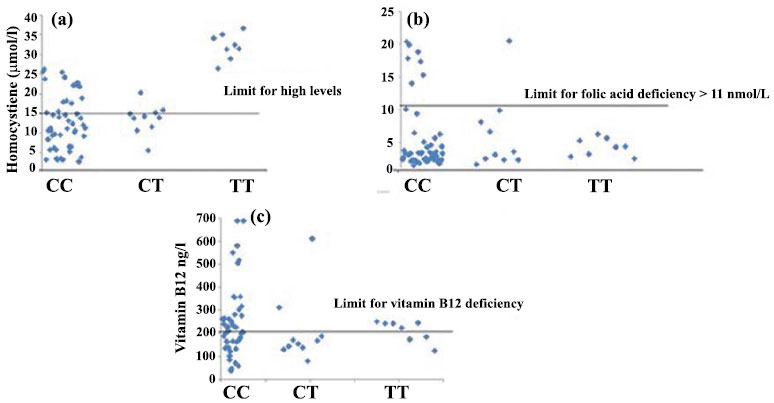
Scatter plots of plasma homocysteine (**a**), folate (**b**) and vitamin B12 (**c**) concentrations in β-TM patients according to their MTHFR genotypes.

**Fig. (5):**
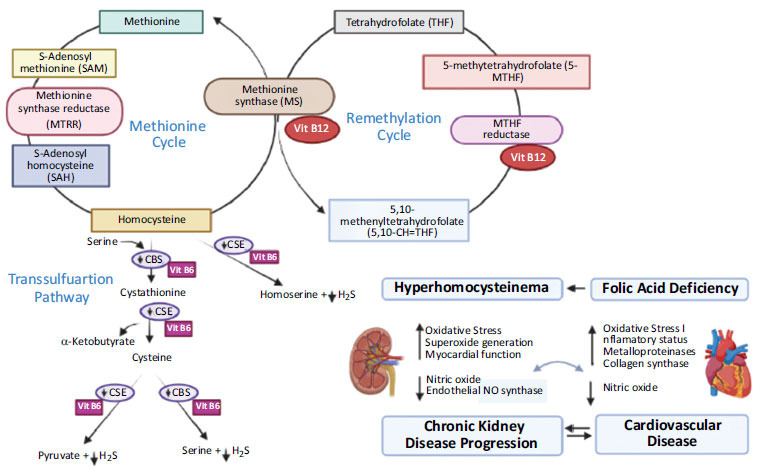
Plasma homocysteine metabolism under physiological and disease conditions: CVD and ESDR.

MTHFR is a gene that may be involved in metabolizing one kind of carbon. Any MTHFR gene mutations coupled with a vitamin B12 deficiency can potentially cause chronic cardiovascular illnesses, which are extremely lethal. The MTHFR C677T polymorphism causes moderate hyperhomocysteinemia, but according to a 1998 study by Brattstrom, the mutation no longer increases the risk of cardiovascular disease [42]. Homocysteine is generated due to the crucial amino acid methionine being demethylated (Fig. **3**). This demonstrates that homocysteine is methylated in the presence of the enzyme methyl synthase, producing methionine [42, 43]. However, folate and vitamin B12 are crucial for the metabolism of homocysteine, which is either generated by the transsulfuration of cysteine or the demethylation of methionine. It should be noted, however, that the methylene-tetrahydro-folate-reductase (MTHFR) enzyme reduces 5,10-methylene THF to 5-methyl THF and only functions when vitamin B12 is present as a cofactor (Fig. **3**). However, Hcy-mediated increased lipid peroxidation and reduced free radicals cause inflammation and acute endothelial abnormalities, hastening the atherosclerotic process that results in cardiovascular disease. Hcy contributes greatly to endothelial dysfunction. Natives of India are extremely likely to have low vitamin B12 levels and hyperhomocysteinemia, especially if they are strict vegetarians or live in cities [7]. Hcy is commonly formed from methionine and is capable of converting back to methionine or cysteine. Hydrogen sulfide can be produced during the metabolism of Hcy in the presence of the enzymes cystathionine b-synthase (CBS) and cystathionine c-lyase (CSE), acting as an angiogenic compound with vasorelaxant and antioxidant properties [42]. Under hyperhomocysteinemia, the depletion of CBS and CSE enzymes takes place, which further causes a reduction in the amount of hydrogen sulfide (H2S), resulting in vascular abnormalities due to the impairment of endothelium. However, recent research suggests that overexpressing the Hhcy metabolizing genes CBS and CSE *via* gene therapy may enhance endothelial functions by producing enough H2S [42]. In terms of the occurrence of Hhcy in patients with β-TM based on their MTHFR genotypes, the data show a significant and clear connection between Hhcy and possession of the MTHFR TT genotype, with all patients with the TT genotype (100%) being assessed hyperhomocystinemic (≥15 mol/l) compared to those with CT (40%) or CC genotypes (29%) P (Fig. **4**). Although it catalyzes the metabolism of methionine, homocysteine (Hcy) is an amino acid typically not involved in protein synthesis. The presence of various parameters, such as the genetic modification of enzymes involved in the metabolism of methionine or the deficiency of vitamins B12, B6, and folate, can be used to determine the concentration of Hcy [44, 45]. In a 2017 research, Juan Ni *et al*. found that blood levels of tHcy and folate (r 14 0.252) and vitamin B12 (r 14 0.243) had substantial negative relationships (*p* 0.001). Compared to women, men showed considerably greater serum tHcy values (*p* 0.001). Compared to those with the CC and CT genotypes of MTHFR, those with the TT genotype had considerably higher blood tHcy concentrations (*p* 0.001). Individuals with the TT genotype had considerably higher folate levels in their red blood cells than those with the CC genotype (*p* 0.05). Additionally, the serum tHcy level was significantly correlated with folate levels (r 14 0.334, *p* 14 0.001) and vitamin B12 (r 14 0.212, *p* 14 0.046) in the low vitamin group. The two main methods for lowering serum Hcy levels are oral FA or 5-MTHF administration or intramuscular injections of FA. However, it should be emphasized that vitamins B12 and B6 are also essential cofactors in the process of metabolizing hydroxy citric acid, in addition to folate [46, 47]. One of the two irreversible mechanisms-transsulfuration to cysteine or remethylation to methionine-can easily degrade Hcy because it can be found close to the center of metabolic processes (Fig. **5**) [46, 47]. Serum tHcy levels were substantially correlated with the MTHFR C677T mutation, folate insufficiency, and B12 deficiency [48]. Folate insufficiency was one of these three variables that had the most impact on the serum tHcy levels, followed by MTHFR C677T and vitamin B12 deficiency (in decreasing order of impact) [48]. So, especially in people with the MTHFR 677TT genotype, folic acid, and vitamin B12 intake may help avoid disorders linked to tHcy buildup. Based on the genotype of MTHFR C677T, the serum level of total homocysteine, vitamin B12, folate, and red blood cell folate, there were significant differences in tHcy concentration between individuals with the TT genotype and those with the CC or CT genotype (*p* < 0.001). Additionally, individuals with the TT genotype had significantly higher RBC folate levels than those with the CC genotype (*p* <0.05) (Table **3B**) [49].

#### 2.2.1 Folic Acid, Vitamin B12, and Hcy: Their Correlation

Dietary B12 and folic acid are closely related. B12 therapy may worsen the neurologic sequelae or insufficiency in patients who are folate-deficient or, conversely, in those who are folate-sufficient. Patients with chronic kidney disease (CKD) have a greater rate of cardiovascular events and death than the general population. The best medical practice advises that cyanocobalamin insufficiency must be excluded before folate supplementation is carried out, or if necessary, it must be suitable to complement folate and diet B12 together in order to avoid treating a B12-poor patient with folate, which exacerbates the neurological condition of both deficiencies [50]. The National Institutes of Health said that excessive folic acid supplementation might conceal the harmful effects of diet B12 deficiency [50]. This issue can be resolved by employing covert laboratory results of megaloblastic anemia, which initially results from vitamin B12 insufficiency. Therefore, medicines that lower Hcy levels may lessen the burden of CVD since Hcy and cardiovascular events are directly correlated. Previous research has shown that folic acid, B vitamins, omega-3 fatty acids, and N-acetylcysteine are the most effective therapy for reducing Hcy levels. Despite having hyperhomocysteinemia, patients with CKD still do not know how Hcy reductions affect their risk of cardiovascular death. Therefore, there are conflicting results in this regard, necessitating further evaluations [50-54]. It is still debatable whether any positive effects of FA treatment should be attributed to its direct effect or a decrease in Hhcy in both the general population and CKD patients [5, 16]. It is possible to think of FA with or without methylcobalamin supplementation as a suitable supplementary treatment in individuals with CKD while awaiting the findings of confirmatory trials.

Table **3A** shows an analysis of sex's effect on hormone concentrations of serum tHcy, vitamin B12, folate, and RBC folate. In men, tHcy concentrations were significantly higher (*p*<0.001), whereas serum folate levels were significantly lower (*p*< 0.05). The folate and vitamin B12 levels in men and women were not different [49].

#### 2.2.2 Health Effects of Food Fortification and Supplement Usage

Supplements have proven to be an efficient source for restoring plasma Cbl levels. Currently, all the government agencies and authorities are equivocal towards a vegan diet comprising LOV, LV, and OV, supplementation of vitamin B12 is necessary [27]. The vitamin B12 per 100 gm dairy products like milk, chicken, eggs, *etc*., ranges from 0.5-0.4μg, from 4.2-3.6 μg, or from 2.5-1.1 μg sequentially [51]. In the HOST trial (Homocysteinemia in Kidney and End Stage Renal Disease), 2056 patients with advanced kidney disease or end-stage renal disease who needed renal replacement therapy and had elevated homocysteine levels were randomized to receive a combination therapy that included folic acid, vitamin B12, and pyridoxine, or a placebo. The study failed to achieve both its primary endpoint, a decrease in all-cause mortality, and its secondary endpoints, a decrease in cardiovascular death, amputations, and thrombosis of the vascular access, even though homocysteine levels had significantly decreased after a median follow-up of 3.2 years. The high cardiovascular comorbidity burden and the inadequate adherence to therapy may be considered potential explanations for these poor results [52-55]. The corresponding quantities are insufficient to provide regular consumption in a stable diet plan when taking cooking losses and absorption rate into account [51]. Furthermore, 85%-100% of patients with end-stage renal disease have hyperhomocysteinemia, which can harm endothelial cells and increase the activity and production of coagulation factors [55-57]. Enalapril with folic acid compared to enalapril had conflicting results on stroke, according to a clinical trial by Arturo J *et al.* published in 2017. Approximately 143 (95% CI 85 to 428) patients would need treatment for 5.4 years to avoid only one stroke [55]. The risk of ischemic heart disease would be reduced by 16% (11% to 20%), deep vein thrombosis by 25% (8% to 38%), and stroke by 24% (15% to 33%), according to meta-analysis research by M. Liu *et al*. published in 2014. This reduction might be achieved by increasing folic acid consumption [58]. In the early stages of CKD, the use of -blockers, renin-angiotensin blockers, diuretics, statins, and aspirin is beneficial [58]. These days, several bacteria, including *Propionibacterium freudenreichii*, *P. shermanii*, and *Pseudomonas dentrificans*, are employed to produce synthetic cobalamin artificially [56]. In some countries, food products like cereals are fortified with Cbl, respectively, for better nutrition profiles among the population. Nevertheless, the quantities used for fortification vary largely, and it does not promise adequacy in the absence of other sources [55]. Cn-Cbl is the most common form of cobalamin since it is highly cost-effective and safe for use [59]. Currently, the accurate upper value for intake of Cbl through supplements is not defined due to the inadequacy of the published data for determining the toxicity events [60]. Elevation in absorption or accumulation of Cbl, Cbl is a water-soluble vitamin that requires a particular transport mechanism that can be easily saturated [60]. The most prevalent cobalamin found in supplement formulations is Cn-Cbl [61]. According to a cohort study by Yujie *et al.* 2022, people with T2D have a significantly higher risk of dying from CVD when their folate and vitamin B12 are low or high [61].

Furthermore, because it maintains stability even at high temperatures, it is particularly suitable for fortification. Provitamin usage is strictly forbidden in individuals with certain hereditary peroxisome-activating enzyme abnormalities [62, 63]. Similar to malabsorption, only around 10 g of large dosages of 1-2 mg are absorbed by non-specific internalization. Because it is less expensive, has fewer side effects, and doesn't hurt when administered, the therapeutic administration of oral Cbl supplements is as effective as intramuscular dosages [63]. Higher dietary folate and vitamin B6 intakes were substantially linked to decreased cardiovascular and all-cause mortality. According to research by Bo *et al.* 2022, consuming more folate and vitamin B6 may reduce the mortality risk for individuals in the United States [35, 63, 64].

### 2.3 Elevated Homocysteine: A Risk Factor for Cardiovascular Diseases

Hyperhomocysteinemia occurs in more than 85% of the total patients suffering from chronic kidney Disease (CKD) due to impairment in their renal metabolism and lowered renal excretion [5]. Various evidence has emerged through recent studies and retrospective research indicating the association of elevated Hcy levels with an increased risk of cardiovascular diseases [65, 66]. It has been studied that homocysteine decreases the biological availability of an essential vasodilator, nitric oxide. However, another mechanism may involve the association of folic acid and homocysteine in the pathology of cardiovascular symptoms known as methoxistasis [65]. Numerous epidemiologic and case-controlled investigations have shown the connection between hyperhomocysteinemia and various consequences, including systemic atherosclerosis, cardiovascular disease (CVD), and stroke. Treating hyperhomocysteinemia with folic acid and B vitamins prevents atherosclerosis, CVD, and strokes. However, later prospective, randomized, placebo-controlled studies have not demonstrated a link between high homocysteine levels or their reduction with therapy and the risk of atherosclerosis, CVD, or strokes, probably due to the folic acid fortification of food [67]. According to cohort research, higher homocysteine levels in Taiwan's middle-aged and senior populations were strongly related to CKD and might be used as a marker of the disease. A higher level of Hcy is also directly linked to a higher risk of cardiovascular diseases like myocardial infarction, which can cause mortality, especially in women over 24, according to research known as the Multi-Ethnic Study of Atherosclerosis (MESA) [68]. The modification in many peripheral disorders is also connected to the rise in Hcy levels, which causes altered flow-mediated endothelium-dependent vasodilation and vasoconstriction, as well as macro and microvascular endothelial repair [69]. In the statin era, Hcy is an independent predictor of cardiac death in patients with stable CAD. Additionally, non-invasive coronary artery imaging supported the role of Hcy in the etiology of CHD. Using electron-beam computed tomography to measure the degree of coronary calcification, it has been found that the relationship between plasma Hcy concentrations and the increase in coronary calcium content is linear [70]. Independent of other risk factors, plasma Hcy levels have been shown to correlate with the degree of coronary artery calcification [71, 72]. According to a study by Ahmad Hassan *et al.* from 2017, in Japanese patients undergoing percutaneous coronary intervention, high Hcy levels before surgery and low HTlase activity were linked to worse long-term mortality. Moreover, regardless of conventional risk variables, Hcy levels are a powerful predictor of death. In this PCI age, this could have effects on risk categorization and treatment strategy [73]. Additionally, it has been demonstrated that the development of coronary plaque load is highly and independently predicted by the presence of prolonged Hcy (>12 l mol/L) [73].

#### 2.3.1 Vitamin B12 Association with Hyperhomocysteinemia

**Fig. (6):**
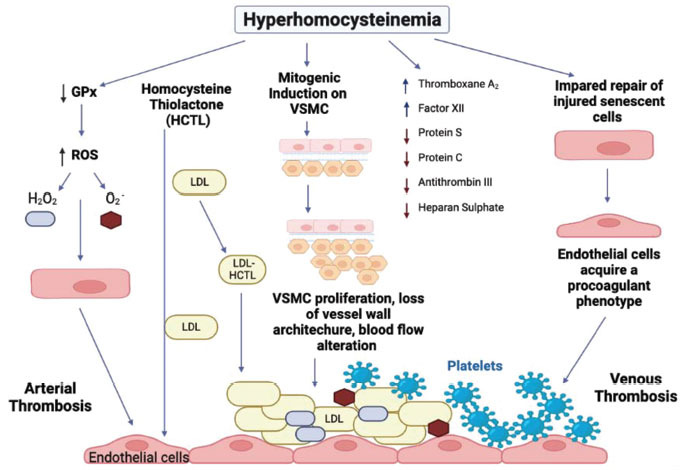
Hyperhomocysteinemia's primary pathogenetic mechanisms for endothelial damage in patients with CVD.

B vitamins, including folate, vitamin B6 and B12, are the major requirements for homocysteine metabolism; various studies have also indicated their potential role in lowering Hcy levels [64]. A few studies indicated the independent role of low doses of vitamin B12 administration as a separate treatment, the reaction of vitamin B12 bio-markers was the primary result, while the tHcy measure was concluded as the experiment's subjects, however, had a slight vitamin B12 deficiency and had not previously received any folic acid supplements [74, 75]. The lack of benefit from vitamin supplementation to lowering homocysteine has called into question the link between moderate hyperhomocysteinemia (HHcy) (15-30 mol/L) and cardiovascular diseases (CVD). Additionally, no dietary component that would have been needed to stimulate the normal mechanism for absorption of vitamin B12 was given along with it. The tHcy lowering impact assessment may have been taken aback by the difference index in folate status or a failure to properly absorb the dosage given [76, 77]. One-carbon (1-C) metabolism requires the proper operation of folate and vitamin B12. The available information, mostly centered on younger or institutionalized populations, is equivocal in terms of relationships between 1-C metabolism biomarkers and mortality. Increased risk of all-cause and CVD mortality in the very old was linked to higher concentrations of tHcy in all participants and plasma vitamin B12 in women. Although the results for tHcy in younger populations are confirmed, the negative associations between elevated plasma vitamin B12 concentrations and mortality in this situation are novel and need more research [78]. Another meta-analysis study made in the USA showed that using vitamin B12 along with folate could produce 1-3 folds more potent effect on lowering tHcy levels than using folate alone [79]. Any additional reduction in tHcy concentration may positively affect CVD risk regarding the link between tHcy and CVD (Fig. **6**). Although, when the subjects considered not receiving any benefit from vitamin B12, *i.e*., those on vitamin B12 supplementation or who were suffering from renal impairment, were excluded from the study, the results among the population showed an enhancement of approximately 21% reduced risk of CVD [78]. Nevertheless, more advanced research should be done regarding the supplementation of vitamin B12 for tHcy lowering to determine the optimal dose (Fig. **6**).

### 2.4 Role of Vitamin B12, Folic Acid and Homocysteine as CVD Risk Markers

Hyperhomocysteinemia has been regarded as a cardiovascular risk marker [46]. Various epidemiologic and case-control-based studies have supported the lineage of elevated Hcy levels with an increased risk of CVD. In contrast, placebo and randomized-based studies do not favor this hypothesis. However, an inconsistency still appears with the role of routine screening of concentration of tHcy and its respective treatment [80]. According to several retrospective investigations, the general public's understanding of homocysteine as a hazard factor is still unclear among subjects with increased homocysteine levels who also have chronic kidney disease (CKD) and end-stage renal disease (ESRD). Moreover, a recent meta-analysis-based retrospective study indicated an elevation in blood homocysteine level as a risk factor for patients with CVD and End-stage Renal Disease ESRD, not treated with any folic acid supplementation [78, 81]. Additionally, research showed that in ESRD patients using supplements, the risk of death and CVD increased by 7% and 9%, respectively, with every significant rise in homocysteine concentration of 5 mmol/L. Data on inverse studies in advanced CKD and dialysis patients were reported, which led to the discovery of a potential phenomenon known as “reverse epidemiology.” Additional CVD risk factors associated with this phenomenon include blood pressure, plasma cholesterol levels, and body mass index [82]. However, data from this research also pointed to a negative correlation between clinical outcomes and Hcy in CKD and ESRD patients. Two studies, in particular, demonstrated that patients with abnormally low homocysteine levels had worse results, as seen by a higher proportion of hospitalization and fatalities [24]. In the young group, hyperhomocysteinemia and CAD were not independently associated risk factors for cardiovascular disease. Measurements of blood homocysteine, folate, and vitamin B12 can be used to assess the risk of hyperhomocysteinemia and juvenile CAD. By taking suitable action, the findings could aid in preventing or delaying the onset of juvenile CAD [24]. In a recent study, Uma A *et al.* 2019 explored the connection between folic acid and vitamin B12 and discovered that it raises mortality overall in patients getting hemodialysis when observed for around five years (9517 patients for the folic acid category and 12,968 patients for the B12 group) [83]. Concluded from the study that greater blood levels of vitamin B12 (550 pg/mL) and lower levels of folate (6.2 ng/mL) may be directly related to the increased risk of all-cause death, either separately or together [77, 84-86]. Additionally, it should be understood that additional dietary modifications for undernutrition, inflammations, and different laboratory and clinical indicators can eliminate the association between folate and morbidity [86].

### 2.5 Effects of Folic Acid and Vitamin B12 Supplementation on CVD and Mortality in CKD and ESRD Patients

The effectiveness of vitamin B12 and folic acid therapy in slowing mortality and end-stage renal disease progression is still unknown. Furthermore, recommendations for people taking supplements of folic acid and vitamin B12-ranging from 2.5 to 5 mg three times per week or even 15 mg daily-have not yet been firmly established [87]. Concurrent administration of the B-vitamin complex has proven to be highly effective in reducing plasma homocysteine levels and re-establishing the demethylation pathway in subjects with ESRD [87, 88]. However, a different trial by Wrone *et al*. found no discernible difference between chronic dialysis patients randomly assigned to 1,5 and 15 mg/day folic acid in terms of mortality or cardiovascular events. According to prospective studies, a 5 mol/L rise in Hcy concentration in ESRD patients has been linked to a 7% increase in the risk of overall mortality and a 9% increase in the likelihood of cardiovascular events. The use of B vitamin supplements in intervention trials appears to have reduced the level of Hcy in these individuals by 13 to 31 mmol/L. Even though mortality had not decreased, this was linked to a 27% decrease in the risk of cardiovascular events [89]. ASFAST (Atherosclerosis and Folic Acid Supplementation Trial) failed to demonstrate the comfort of folic acid therapy against all forms of mortality in patients with CKD and ESRD and controlling atheroma progression. According to a meta-analysis by Yuan W *et al*. in 2019, patients with CVD may experience a significantly lower risk of stroke when taking folic acid supplements [90]. It was made with a placebo cohort of 315 subjects, but the results showed no significant benefit regarding folic acid treatment in patients with CKD dialysis. Homocysteine level determination in Kidney and End Stage Renal Disease was a double-blind, placebo-controlled clinical experiment in which 2056 patients who needed urgent renal replacement therapy and a rise in plasma homocysteine levels were given randomized supplements of folic acid, vitamin B12, and pyridoxine [90, 91].

### 2.6 Vitamin B12 and Cardiovascular Diseases: A Preventive Measure

Vitamin B12 aids in the metabolism of homocysteine to methionine. Therefore, the elevated vitamin B12 among the population can be beneficial for patients with increased Hcy levels, reducing the risk of strokes [92]. Although increased plasma homocysteine concentrations are a well-known risk factor for cardiovascular disease, it is unclear whether these concentrations act as standalone biomarkers for the disease process or as modifiable risk factors. By taking supplements and consuming foods high in folic acid and vitamin B12, Hcy levels can be decreased reasonably, lowering the risk factor for CVD [92, 93]. The average CVD risk is reduced by 7% with vitamin B12 supplementation (0.02-1 mg/d), while the risk can be reduced by 10% to 30% with folate, respectively. Endothelial impairment is an early risk marker for atherogenesis and vascular disease progression. Although improving endothelial functioning can further prevent the risk of CVD [94]. Folate can improve endothelial functions in coronary artery diseases through a mechanism that does not affect the Hcy level [54]. Although the impact of supplementing with vitamin B12 alone against CVD has not yet been studied, it has been demonstrated that doing so can lessen homocysteine levels when combined with folic acid [95, 96]. Vitamin B12 has resulted in having a lesser effect when compared with folate supplementation. According to research, supplementing subjects with 5 mg of folic acid and 250 mg of vitamin B12 for 12 weeks reduced plasma homocysteine levels by 32% in subjects with coronary artery disease [55]. Another clinical trial involving healthy men and women between the ages of 70 and 90 found that regular supplementation with 500 mg of vitamin B12, 0.8 mg of folate, and 3 mg of vitamin B6 may be able to reduce serum homocysteine levels by 0.001. This research implies that homocysteine levels rise even in healthy individuals due to vitamin deficits (Table **4**) [97-100].

## 3 DISCUSSION

Currently, the existing trial data does not fully support using FA and vitamin B12 levels as valid indicators of CVD risk in the CKD and ESRD population. A limiting risk factor for detecting any major effects of vascular disease is often pointed out as a very low level of blood Hcy concentration and an already quite high folate level. However, increased tHcy levels and much lower levels of B vitamins, important for the metabolism of homocysteine, confirmed by the research mentioned above showed that type 2 diabetes patients are at risk for developing early cardiovascular disease. However, it is yet unknown how homocysteine raises the risk of CVD and the deaths that accompany it. An important risk factor for cardiovascular disease (CVD) that can be removed with vitamin B12 and folic acid supplementation is the hypothesis that any major folate-sensitive abnormality in homocysteine metabolism may lead to diminished methylenetetrahydrofolate reductase activity Patients with mild to moderate CKD and higher B12 levels who received folic acid treatment saw a greater decline in their risk of CKD progression. Nevertheless, there is some evidence to suggest that the incidence of stroke and the progression of CKD may be controlled using more focused FA therapy (baseline FA levels may affect the effectiveness of the FA intervention therapy), particularly in people with the MTHFR 677TT genotype, low to moderate levels of folate, and in nations without a grain fortification program. Plasma homocysteine (tHcy) as a cardiovascular risk factor is a topic of considerable research. However, current developments in its prevention are more likely to support the value of tHcy-lowering treatment with vitamin B12. Nevertheless, it is still unclear whether the positive effects of FA treatment in the general population and in CKD patients result from a direct antioxidant impact or a decrease in HHcy. In the examined research, inconsistent outcomes in CKD progression and cardiovascular risk are caused by variations in patient characteristics and FA treatment plans between trials. They may be impacted by the severity of cardiovascular and renal impairment.

## CONCLUSION

Folic acid is a suitable supplementary therapy for patients with CVD, CKD, and ESRD receiving treatments, whether or not they also get vitamin B12 supplementation. In these circumstances, FA may be supplied pharmacologically following a careful assessment of folate status.

## Figures and Tables

**Fig. (1):**
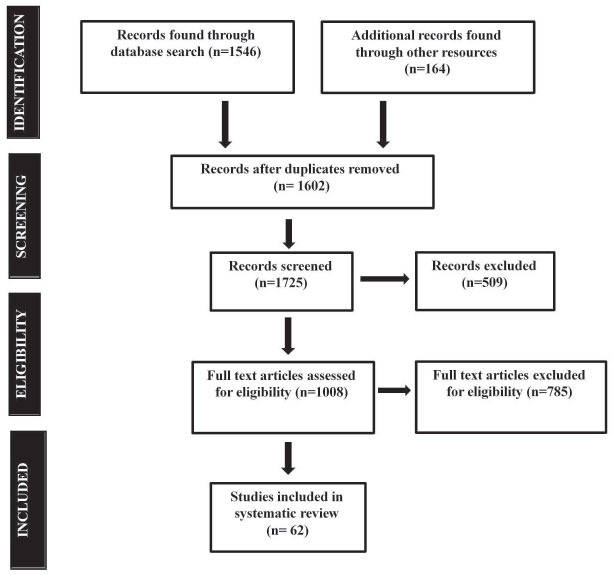
Flow diagram to illustrate selection criteria for the review on Homocysteine, vitamin B12 and folate level: as potential risk factors in the progression of chronic heart and kidney disorders.

**Table 1:** Comparison of subjects with homocysteine plasma levels that are equal to or higher than 15 mol/L to those with homocysteine plasma levels those are lower than 15 mol/L.

-	**All subjects**	**Men**	**Women**
**Model 1**	8.30 (6.17 - 11.16)	6.93 (4.97 - 9.67)	14.89 (7.44 - 29.81)
**Model 2**	7.59 (5.53 - 10.41)	6.98 (4.96 - 9.82)	10.44 (5.05 - 21.55)
**Model 3**	7.43 (5.41 - 10.21)	6.83 (4.84 - 9. 62)	10.26 (4.97 - 21.20)

**Note:** Model 1: crude

Model 2: according to age

Model 3: correlated with age, smoking status, body mass index, hypertension, and diabetes mellitus

**Table 2:** Comparison of homocysteine plasma levels equal.

-	**Men**	**Women**
**Model 1**	5.76 (3.81 - 8.72)	11.08 (4.30 - 28.54)
**Model 2**	4.82 (3.16 - 7.35)	7.16 (2.75 - 18.60)
**Model 3**	4.93 (3.21 - 7.58)	7.03 (2.69 - 18.37)

**Note:** Model 1: crude

Model 2: adjusted for age

Model 3: adjusted for age, smoking status, body mass index, high blood pressure, and diabetes mellitus

Researchers calculated the odds ratio (95% confidence interval) of having CKD.

**Table 3A:** Influence of sex on total homocysteine, vitamin B12, folate, and red blood cell folate levels.

**Sex**	**tHcy (mM)**	**B12 (pM)**	**Folate (nM)**	**RBC Folate (nM)**
Men (n 1⁄4 164)	15.77 ± 8.80	416.18 ± 189.69	19.46 ± 14.12	674.91 ± 365.92
Women (n 1⁄4 166)	11.77 ± 6.32	446.74 ± 216.70	22.99 ± 13.84	737.01 ± 427.62
Total (n 1⁄4 330)	13.76 ± 7.90	431.55 ± 203.99	21.24 ± 14.07	706.15 ± 398.76

**Table 3B:** The serum levels of total homocysteine, vitamin B12, folate, and red blood cell folate based on the genotype of MTHFR C677T.

**Genotype**	**tHcy (mM)**	**B12 (pM)**	**Folate (nM)**	**RBC Folate (nM)**
CC (n 1⁄4 146)	11.69 ± 3.87	439.81 ± 202.81	21.86 ± 13.97	648.14 ± 385.20
CT (n 1⁄4 136)	13.32 ± 5.50	426.40 ± 201.35	21.81 ± 14.85	731.22 ± 388.00
TT (n 1⁄4 48)	21.28 ± 15.22	421.03 ± 218.01	17.74 ± 11.65	811.54 ± 446.41

**Table 4:** Impact of vitamin B12 on human health.

**Vitamin B12 Related Diseases**	**Description**	**Age Group**	**Possible Treatments**	**References**
Chronic atrophic gastritis	Chronic atrophic gastritis (CAG) is one of the major consequence of an inflammatory effect that ultimately results in lack of suitable mucosal glands. Destruction of parietal cells, both autoimmune-driven or due to Helicobacter pylori contamination, determines reduction or abolition of acid secretion.	Late adult hood or around 50-80 years of age group	Vitamin B12 injections and supplements, folic acid supplements, medicines, antibiotics	[14, 36, 57]
Acute myocardial infraction	According to latest finding, it has been clinically confirmed that decrease attention of pyridoxal phosphate is notably related to extended chance of myocardial infarction in predominantly post- menopausal ladies. A excessive consumption of folates, nutrition B12 and their aggregate is inversely related to AMI chance.	The mean age of AMI is 55.9 to 62.9	Nutritional therapy, intravenous heparin infusion, percutaneous coronary intervention (PCI) ticagrelor, reperfusion, aspirin, high-dose statin, blocker, ACE-inhibitor	[37-39, 58]
Heart strokes	Nutrition is highly critical in stroke threat than most physicians suppose. Healthy life-style reduces the danger of stroke. Patients prone to stroke need to maintain their salt consumption to 2-3 grams according to day. They need to additionally have metabolic B12 deficiency and hyperhomocysteinemia detected and treated, possibly with methyl cobalamin.	Mean age in women is 73.2 and in men it is 68.1	IV injection of recombinant tissue plasminogen activator (TPA), alteplase (Activase) or tenecteplase (TNKase), endovascular therapy, stent retriever, caratid endarterectomy, angioplasty	[40, 41, 50]
Chronic heart failure	Patients with HF have many comorbidities, which may also bring about acute decompensation. Moreover, malabsorption because of edema of the intestinal mucosa and liver disorder can result in malnutrition. Consequently, among subjects which frequently have iron, vitamin B12, and/or folic acid deficiency are more prone to HF.	Persons aged 60 years or older	Drug based (Angiotensin-converting enzyme (ACE) inhibitor, drugs, angiotensin II receptor blocker drugs, β-blocker drugs, Diuretics, spironolactone), coronary bypass surgery, heart valve repair, implantable cardioverter-defibrillators (ICDs), cardiac resynchronization therapy, ventricular assist devices, heart transplant	[42, 43, 60]
Congenital heart defects	Women planning a pregnancy and pregnant ladies withinside the first trimester are encouraged to use folate-containing dietary supplements so that they can prevent neural tube defects. Additionally, toddlers and kids with congenital coronary heart defects regularly display issues in folate metabolism (low folate, better homocysteine, or low nutrition B12).	Generally diagnosed between 0 to 5 years of age	Medication (blood pressure drugs, diuretics, heart rhythm drugs), surgery (cardiac catheterization, heart surgery, heart transplant, fetal cardiac intervention)	[44, 45, 59]
Atherosclerosis	High-dose B-nutrition supplementation considerably reduces development of early degree subclinical atherosclerosis (carotid artery intima-media thickness) in well-nourished wholesome B-vitamin “replete” people at low-threat for CVD with a fasting tHcy >9.1 μmol/L.	Generally occurs during adulthood, 20-35 years of age	Medication (statins and cholesterols drugs, aspirin, blod pressure medication), surgery (angioplasty and stents placement, endarterectomy, fibrinolytic therapy, coronary artery bypass graft (CABG))	[46-49, 56]
Cerebrovascular diseases	In cases of heart diseases and strokes, a more detailed understanding of homocysteine metabolism is necessary, especially in cases of elevated Hcy level. However, supplementing vitamins in order to reduce Hcy level, has not proven to be effective as a secondary prevention in case of myocardial infractions, stokes and mortality in case of cardiovascular diseases.	Most common in people aged 65 or above	Carotid artery surgery, computer assisted surgery, craniotomy, embolization, Encephaloduroateriosynangiosis (EDAS), endovascular aneurysm treatment, endovascular neurosurgery, gamma knife radiosurgery, microsurgery, stenting aneurysm clipping	[11, 16]
Cancer	DNA repair and synthesis is very crucial role of folic acid. Ames, suggested that deficiencies of B vitamin complexes specially folate, vitamin B12 and B6, is associated with increased risk of cancer, due to incorporation of uracil in place of appropriate base into human DNA may resulting in chromosomal abruptions.	Median age of cancer diagnosis is 66 years	Supplementation of folic acid and vitamin B12, chemotherapy, hormone therapy, hyperthermia, immunotherapy, photodynamic therapy, radiation therapy, stem cell transplant, surgery, targeted therapy	[13, 16]
Mental health	Researchers have hypothesised the role of homocysteine is not only associated with cardiovascular diseases but also with other brain related disorders. Furthermore, the concentration of plasma Hcy alone, is a risk factor in declining cognitive performance among the general ageing population irrespective of vitamin B12 concentrations.	Highly prevalent among youngsters, young adults aged 18-25 years	Medication (antidepressants, anti-anxiety medication, mood-stabilizing medicines, antipsychotic drugs), psychotherapy, brain-stimulation treatment, substance misuse treatment, meditation, vitamin B12 and folate supplementation	[16, 24]
Osteoporosis	Major dietary factors associated with the progression of osteoporosis among natives involves; insufficient calcium, proteins and vitamin D. More recently, a study has revealed the association of other nutrients such as vitamin B12 on bone health. Moreover, a positive result has been found by supplementing vitamin B12 on subjects with low Bone Mineral Density (BMD), in osteoporosis patients with elevated Hcy level and CVD patients with risk of osteoporosis.	From about age 25 to age 50	Bisphosphonates [alendronates, ibandronate, risedronate, zoledronic], denosumab [prolia, Xgeva], hormone -related therapy (estrogen), bone-building medications [teriparatide, abaloparatide, romosozumab]	[11]

## LIST OF ABBREVIATIONS

CVD: Cardiovascular Disease
ASFAST: Atherosclerosis and Folic Acid Supplementation Trial
ESRD: End-Stage Renal Disease
CKD: Chronic Kidney Disease
HHcy: Hyperhomocysteinemia
MTHFR: Methylenetetrahydrofolate reductase
